# **Improved cardiovascular health by supplementation with selenium and coenzyme Q10**: **applying structural equation modelling (SEM) to clinical outcomes and biomarkers to explore underlying mechanisms in a prospective randomized double-blind placebo-controlled intervention project in Sweden**

**DOI:** 10.1007/s00394-022-02876-1

**Published:** 2022-04-06

**Authors:** Urban Alehagen, Peter Johansson, Erland Svensson, Jan Aaseth, Jan Alexander

**Affiliations:** 1 Division of Cardiovascular Medicine, Department of Health, Medicine and Caring Sciences, Linköping University, 581 85 Linköping, Sweden; 2 Department of Health, Medicine and Caring Sciences, Linköping University, 601 74 Norrköping, Sweden; 3 Swedish Defence Research Agency, Stockholm, Sweden; 4 Research Department, Innlandet Hospital Trust, 2381 Brumunddal, Norway; 5 Faculty of Health and Social Sciences, Inland Norway University of Applied Sciences, 2418 Elverum, Norway; 6 Norwegian Institute of Public Health, 0403 Oslo, Norway

**Keywords:** Selenium, Coenzyme Q10, Elderly, Mechanisms, Mortality, SEM

## Abstract

**Purpose:**

Selenium and coenzyme Q10 have synergistic antioxidant functions. In a four-year supplemental trial in elderly Swedes with a low selenium status, we found improved cardiac function, less cardiac wall tension and reduced cardiovascular mortality up to 12 years of follow-up. Here we briefly review the main results, including those from studies on biomarkers related to cardiovascular risk that were subsequently conducted. In an effort, to explain underlying mechanisms, we conducted a structured analysis of the inter-relationship between biomarkers.

**Methods:**

Selenium yeast (200 µg/day) and coenzyme Q10 (200 mg/ day), or placebo was given to 443 elderly community-living persons, for 48 months. Structural Equation Modelling (SEM) was used to investigate the statistical inter-relationships between biomarkers related to inflammation, oxidative stress, insulin-like growth factor 1, expression of microRNA, fibrosis, and endothelial dysfunction and their impact on the clinical effects. The main study was registered at Clinicaltrials.gov at 30th of September 2011, and has the identifier NCT01443780.

**Results:**

In addition to positive clinical effects, the intervention with selenium and coenzyme Q10 was also associated with favourable effects on biomarkers of cardiovascular risk. Using these results in the SEM model, we showed that the weights of the first-order factors inflammation and oxidative stress were high, together forming a second-order factor inflammation/oxidative stress influencing the factors, fibrosis (β = 0.74; *p* < 0.001) and myocardium (β = 0.65; *p* < 0.001). According to the model, the intervention impacted fibrosis and myocardium through these factors, resulting in improved cardiac function and reduced CV mortality.

**Conclusion:**

Selenium reduced inflammation and oxidative stress. According to the SEM analysis, these effects reduced fibrosis and improved myocardial function pointing to the importance of supplementation in those low on selenium and coenzyme Q10.

## Background

Selenium and coenzyme Q10 were supplemented in a four-year intervention study, to an elderly population (*n* = 443) with a low selenium status and because of old age, a low level of coenzyme Q10. A significantly reduced cardiovascular mortality was observed after five, 10 and 12 years [1–3]. Here we present an overview of the intervention trial including clinical outcomes and secondary analyses conducted mainly on different biomarkers. Second, we present a model for the interconnection between biomarkers and clinical outcome obtained by means of Structural Equation Modelling (SEM) analyses. With the application of SEM analysis, which is a combination of factor and multiple regression analyses, it is possible from observed or manifest variables to find underlying or latent factors explaining the relations between the observed variables, as well as to estimate the effects of the latent variables on dependant variables. Latent variables or factors provide important information, e.g. on pathophysiological processes, which cannot be fully described by single biomarkers alone.

Previously, in an attempt to explore possible underlying mechanisms, we have undertaken a series of secondary analyses, examined variables, such as changes in biomarkers for myocardial wall tension, inflammation, oxidative stress and fibrosis, as well as impact of baseline selenium status that all could be related to the outcome presented in the main study. A limitation, however, is that these studies by themselves do not present a broader perspective of how supplementation with selenium and coenzyme Q10 can be associated to changes in basic physiological mechanisms and how alterations in these mechanisms can be associated with each other. In the present paper, we first briefly summarize our previous results and then conduct a structural equation modelling analysis leading to an overall description of possible mechanisms and observed clinical effects.

## Selenium and coenzyme Q10 in humans

Selenium is a trace element that is essential for a normal cellular function, and several of the selenoproteins play crucial roles in the cellular red/ox regulation and the protection against oxidative stress. To obtain an optimal expression of one of the important selenoproteins in plasma; selenoproteins P, which transports selenium from the liver to peripheral tissues, a daily intake of 100–150 µg/day of selenium is required [4]. Of specific interest among the 25 selenoproteins is that the thioredoxin reductases (TXRND), a group of selenoproteins that belongs to the pyridine nucleotide–disulfide oxidoreductase family, which is central to the cellular redox regulation, appear to require at least as high serum selenium levels as other human selenoenzymes to be optimized (i.e. fully expressed), apparently about 120 µg/L when measured in erythrocytes [5].

However, the content of selenium soil varies in different parts of the world, influencing the daily intake. In Europe, the concentrations of selenium in plasma are generally low, usually well below 80–90 µg/L [6–9], whereas in North America, the levels are generally above 120 µg/L [10, 11]. In Finland, the average intake level of selenium in the 1970s was 25 µg/day with a corresponding plasma selenium concentration of 50 µg/L [12]. After the decision to add selenium to the fertilizers in 1986, the average daily intake has risen to about 80 µg/day [12].

The French EVA study demonstrated an association between low plasma concentration of selenium and increased mortality [13]. However, in a meta-analysis including 12 different trials (including the American SELECT and NPC trials), Rees et al. could not find an association between supplementation with selenium and decreased cardiovascular mortality [14]. It is, however, important to note that both the SELECT and NPC studies included US participants with mean plasma selenium concentrations higher than in all European countries (135 μg/L, and 113 μg/L respectively) [15], and that the vast majority of the participants in the meta-analysis reported by Rees et al. were US inhabitants from the SELECT and NPC studies.

In Sweden, there is situation similar to that which existed in Finland before selenium was added to fertilizers, with low selenium content in the soil resulting in a low daily intake as reported by Harris et al. demonstrating an intake of less than 25 µg/day [16], and by Gao et al. who found the intake to be well below the recommended dietary intake of selenium [17]. However, as many studies were based on dietary surveys, or were performed several decades ago, we wanted to obtain new data based on analyses in blood samples from a representative elderly community-living population. A total of 668 elderly participants were included from a rural municipality population in the Southeast of Sweden [18]. The study population showed a low mean serum selenium concentration, 67.1 µg/L. This corresponds to an average daily intake of approximately 35 µg/day, thus well below the amounts (110–150 µg/day) for an optimal expression of selenoproteins [19]. The consequence of low selenium status in terms of cardiovascular (CV) mortality was evaluated in our Swedish study population during a follow-up period of 6.85 years [20]. A significantly higher CV mortality was observed among participants in the lowest quartile (29 out of 107) compared with those in the highest quartile (16 out of 111; χ^2^: 5.35; *p* = 0.02). In a multivariate model where adjustments were made for male gender, smoking, ischaemic heart disease (IHD), diabetes, chronic obstructive pulmonary disease, and impaired systolic cardiac function (EF < 40%), the increased CV mortality risk remained significant in the lowest selenium concentration quartile (HR: 1.56; 95%CI 1.03–2.36; *p* = 0.04), compared with those who had a higher selenium intake. Thus, it seems that a low daily intake of selenium has important implications for human health, which is also reported in the literature [21, 22].

However, of importance, as reported by Xia et al., coenzyme Q10 needs the presence of selenium for reduction of the oxidized form, ubiquinone, into the active form, ubiquinol [23]. Coenzyme Q10 is essential for all living cells, and it is one of the important antioxidants in lipid structures — membranes and lipoproteins of the body and of particular importance for crucial ATP-generating steps within the mitochondrial respiratory chain. In the extramitochondrial space, the selenoenzyme TXNDR1 is a main reductive enzyme restoring ubiquinol from ubiquinone [23]. The endogenous production of coenzyme Q10 decreases with increasing age, and the production in the myocardium is reduced to about half at the age of 80 years [24], why supplementation to elderly, especially for those living in areas with low selenium contents in the soil seems relevant [24, 25]. Both the synthesis of coenzyme Q10 and selenoproteins, in this case TXNDR is dependent on a functional mevalonate pathway [25].

These considerations suggest that optimal levels in the organism of *both* selenium and coenzyme Q10 are needed for good human health.

Based on the rationale outlined above, a group of elderly Swedish individuals was recruited into a prospective randomized double-blind placebo-controlled trial. The study population included consisted of 443 individuals in the age 70–88 years (Table 1). They received the selenized yeast and coenzyme Q10 (SelenoPrecise 200 µg B.I.D, Pharma NordApS, Vejle, Denmark and Bio-Quinon 100 mg B.I.D, Pharma NordApS, Vejle, Denmark) or placebo as a dietary supplement for 48 months and were re-examined every six months [1].

**Table 1:** Baseline characteristics of the study population receiving dietary supplementation of selenium and coenzyme Q10 combined or placebo during 4 years

	Active treatment group *N* = *221*	Placebo group *N* = *222*	*P*
Males/Females *n*	115/106	110/112	
Age years, mean (SD)	77.0 (3.6)	77.3 (3.4)	0.36
*History*			
Diabetes, *n* (%)	47 (21.3)	48 (21.6)	0.93
Hypertension, *n* (%)	158 (71.5)	168 (75.7)	0.32
IHD, *n* (%)	47 (21.3)	53 (23.9)	0.51
NYHA class I, *n* (%)	118 (53.4)	108 (48.6)	0.32
NYHA class II, *n* (%)	61 (27.6)	64 (28.8)	0.77
NYHA class III, *n* (%)	41 (18.6)	47 (21.2)	0.49
NYHA class IV, *n* (%)	0	0	
NYHA unclassified, *n*	0	3	
*Medications*			
ACEI, *n* (%)	35 (15.8)	55 (24.8)	0.02
ARB, *n* (%)	10 (4.5)	13 (5.9)	0.53
Beta blockers, *n* (%)	81 (36.7)	73 (32.9)	0.40
Digitalis, *n* (%)	11 (5.0)	11 (5.0)	0.99
Diuretics, *n* (%)	70 (31.7)	88 (39.6)	0.08
Statins, *n* (%)	45 (20.4)	51 (23.0)	0.50
*Examinations*			
EF < 40%, *n* (%)	16 (7.2)	17 (7.7)	0.87
Atrial fibrillation, *n* (%)	24 (10.9)	25 (11.3)	0.89
NT-proBNP, ng/L, mean (SD)	547 (1578)	517 (877)	0.81

Note: *ACEI* ACE inhibitors, *ARB* Angiotensin receptor blockers, *EF* Ejection fraction, *IHD* Ischemic heart disease, *NT*-*proBNP* N-terminal fragment of proBNP, *NYHA* New York Heart Association functional class, *SD* Standard Deviation

Note: Values are means ± SDs or frequency (percent)

Note: Student’s unpaired two-sided t test was used for continuous variables and the chi-square test was used for analysis of one discrete variable

## Summary of effects on primary and secondary outcomes

### Glycation products

**Fig. 1:**
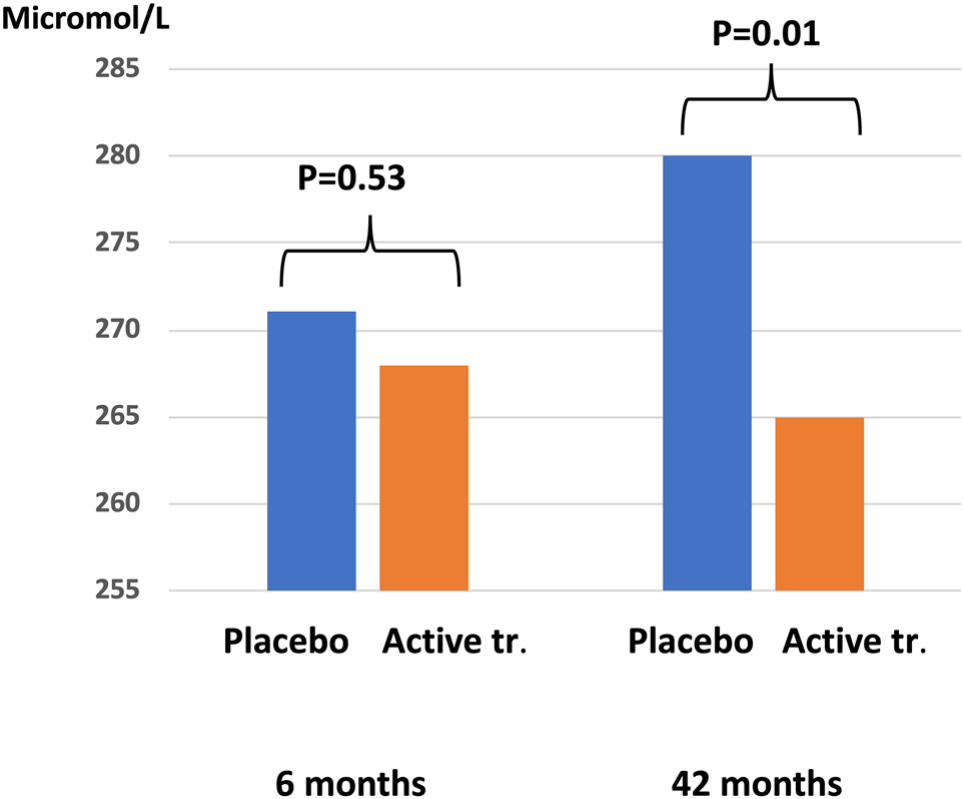
Plasma concentration of fructosamine in those with active treatment compared with placebo after six and 42 months

Fructosamine is the glycation product of proteins, mainly albumin, in the body. The concentration of fructosamine monitors the blood–glycose level during the last two to four weeks. However, fructosamine appears to be associated with other pathogenic mechanisms in the body. Even in those without diabetes, a higher concentration of fructosamine indicates an increased CV risk [26]. Also, there is a strong association between concentration of fructosamine and biomarkers of inflammation and increases in patients with rheumatoid arthritis, but without diabetes [27]. Similarly, in the non-diabetic part of the study population, we found a strong association between the fructosamine concentration and intervention with selenium and coenzyme Q10, as a significantly decreased fructosamine concentration was observed in the active treatment group (Fig. 1) [28].

The effect on fructosamine could be seen in both genders, and both in the older, and in the younger part of the study population. This indicates that an anti-inflammatory effect i.e. of selenium also may protect against protein glycation, which has previously been discussed in the literature [29].

### Insulin-like growth factor 1 (IGF-1)

Insulin-like growth factor 1 (IGF-1) has a multitude of actions, including anti-inflammatory and anti-apoptotic actions on myocardial cells [30, 31].The concentration of IGF-1 decreases with increasing age [32]. A lower concentration of IGF-1 is also associated with increased CV risk [33, 34]. In our study, we observed higher levels of both IGF-1, and the age-adjusted IGF-1 SD score as a result of the intervention [35].

These increased IGF-1 concentrations accompanying the intervention could mediate positive intracellular effects including less inflammatory activity and probably also an anti-apoptotic effect.

### Inflammation

Several selenoproteins have important functions in the inflammatory response accompanying various diseases [36]. To evaluate the effects of supplementation with selenium and coenzyme Q10 on inflammation, we used sP-selectin and CRP using high sensitivity assays. We could report a highly significant difference after 48 months of both biomarkers, with a lower concentration in those supplemented with selenium and coenzyme Q10 [37]. The effect appears to be partly mediated by p38 mitogen-activated protein kinases or c-JNK signalling pathways [38, 39].Therefore, it seems reasonable that the supplementation of selenium and coenzyme Q10 has effects also on inflammation.

To further validate these results, five additional biomarkers of inflammation (osteopontin, osteoprotegerin, sTNF receptor 1, sTNF receptor 2 and the tumour necrosis factor-like weak inducer of apoptosis called TWEAK) were examined [40].

Significant differences were observed in four out of five of the biomarkers, with signs of less inflammatory activity in those on active supplementation with selenium and coenzyme Q10, indicating less inflammatory activity in those supplemented with selenium and coenzyme Q10, as compared to those on placebo.

### Oxidative stress

Several selenoproteins and coenzyme Q10 are both important endogenous antioxidants [41, 42]. The reduced form of coenzyme Q10, ubiquinol, protects against lipid peroxidation, and together with selenium, it can reduce the inflammatory response, as discussed above [43, 44].

**Fig. 2:**
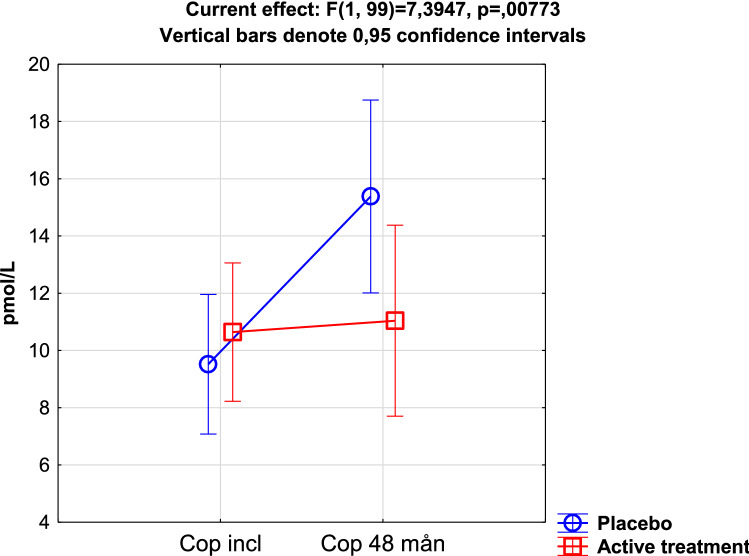
Plasma concentration of copeptin in the active treatment group, compared with placebo at inclusion and after 48 months

With increasing age, the level of oxidative stress is increased [45–47]. Copeptin, a surrogate biomarker for vasopressin, and the biomarker adrenomedullin have both been shown to act as markers of the level of oxidative stress in tissues [48, 49]. In those on supplementation with selenium and coenzyme Q10, the levels of the two biomarkers decreased in comparison with those on placebo[50] (Fig. 2). This indicated a reduced intracellular oxidative stress due to the intervention.

### Endothelial function

Endothelial function and selenium concentration are associated, and this might have clinical implications [51, 52]. Therefore, two biomarkers of endothelial function were investigated, the von Willebrand factor, and plasminogen activator inhibitor.

The von Willebrand factor is a plasma glycoprotein that is primarily active in the coagulation process, through a process in the vascular endothelium [53], but also an indicator of endothelial dysfunction [54].

Plasminogen activator inhibitor (PAI-1) is secreted from the endothelium and hepatocytes [55, 56], has well-known antifibrinolytic effects. The factors are also indicators of the endothelial function.

Our studies disclosed a highly significant difference between active treatment and placebo after 36 months with significantly higher concentrations of the two biomarkers in the placebo group, compared with the active treatment group [57], indicating that the intervention results in less endothelial dysfunction. This could indicate less injury of the vascular endothelium, an effect that later could be manifested as established CV disease, and where the end-result could result in a CV death.

Finally, D-dimer, is a fragment of degraded fibrin, and as an indicator of fibrinolysis [58], it is used as a biomarker for venous thromboembolism [59], or pulmonary embolism [60]. However, there is also an association between concentration of D-dimer and mortality risk, even in the absence of thromboembolism, possibly because D-dimer is also a biomarker of inflammation [61]. In addition, there may be an association between D-dimer and endothelial dysfunction as well [62]. In our study, a significantly lower concentration of D-dimer could be noted in the supplementation group, compared with those on placebo [63].

### Fibrosis

In the process of ageing and development of diseases in the CV system, fibrosis is one of the characteristics observed [64]. Eight different biomarkers for fibrosis (endostatin, tissue metalloproteinase 1 (TIMP1), galectin 3, and growth differentiation factor 15 (GDF-15), cathepsin S, matrix metalloproteinases 1 and 9 (MMP-1, MMP-9) and Suppression of tumorigenicity 2 (ST2)) have been evaluated. From these evaluations, significant reductions of concentrations in seven of the eight biomarkers were observed. This could imply that the intervention influenced the fibrous process in the CV system with less fibrosis as a result.

### microRNA

The knowledge of microRNAs is fairly recent, and, today, more than 2000 different microRNAs have been characterized [65, 66]. The microRNAs have important functions in the regulation of the protein-encoding mammal genes, and one single microRNA can regulate the production of many different proteins. We found that the combined supplementation resulted in a highly significant difference in expression in 90 out of 145 microRNAs and with up to a fourfold change, when comparing those to placebo [67]. Among these, several microRNAs were related to CV disease, myocardial repair and myocardial function. Substantial changes in microRNA expression observed may act to modulate the synthesis not only of the selenoproteins, but possibly also of other proteins.

### Cardiac function

Heart failure is characterized by increased wall tension that leads to increased synthesis of the natriuretic peptide BNP and its N-terminal fragment of proBNP (NT-proBNP). The most important effect of the release of BNP is increased diuresis, and vascular dilatation. However, besides heart failure, increased age is also associated with increased synthesis of the peptide.

Supplementation with selenium and CoQ10 resulted in a decreased NT-proBNP concentration, when compared with the placebo group, indicating reduced myocardial wall tension [1]. The decrease in NT-proBNP is also in accord with a significantly improved systolic cardiac function in the active treatment group as measured by change in ejection fraction (EF) as seen in echocardiography [1].

### Cardiovascular mortality and quality of life

The intervention resulted in a significantly reduced CV mortality (5.9% in the active supplementation group versus 12.6% in the placebo group; *p* = 0.015), by applying a follow-up time of five years, and a corresponding CV mortality risk reduction in those supplemented with selenium and coenzyme Q10, was found.

By applying a longer follow-up period, 10 years, still a significant reduction of CV mortality remained (46 out of 221 in the active treatment group compared with 86 out of 222 in the placebo group; *p* < 0.0001), and with a corresponding risk reduction, apparent also after adjustment for several well-known variables influencing CV risk [2]. In addition, the positive long-term results have been validated in a follow-up evaluation after 12 years with a HR of 0.59 (95%CI 0.42–0.81; *p* = 0.001) of CV mortality risk when using a multivariate model [3]. It should be emphasized that the active intervention was terminated already after four years.

Furthermore, in the project, the health-related quality of life has been evaluated by use of the generic health-related quality of life questionnaire SF-36. The result was a better quality of life reported both in the physical and in the emotional dimensions compared to those on placebo supplementation, which support the results on positive effects combined supplementation discussed above [68].

### Aim of present analysis

The aims of the present secondary analysis were to use clinical and biomarker results summarized above to further explore underlying mechanisms behind the reduced CV mortality and improved cardiac function in the elderly Swedish population that was supplemented with selenium and coenzyme Q10, by the use of the structural equation modelling (SEM) technique, and to elucidate cause–effect relations of the supplementation.

## Methods of the present secondary analysis

The previously presented main study and the summarized sub-studies are based upon the classical experiment-control group design (a “randomized double-blind placebo-controlled trial”), whereas the present secondary analysis applying a SEM-analyses approach, represents an alternative analytical technique for causal inferences. The SEM analyses also represent a cross-validation of the findings from the main study. Our research group has used SEM modelling in previous publications [69, 70].

### Statistical method: structural equation modelling analysis

A stepwise approach in the analysis was adopted.

In a first step, Confirmative Factor Analysis (CFA) was used to reduce the complexity of the 13 measured biomarkers into a lower number of latent, underlying variables or factors. The choice of variables to be measured is central in the evaluation process. However, from the previously published sub-analyses, we could see that obviously some mechanisms were influenced to a greater extent than others. We could see that inflammation, oxidative stress, and fibrosis were examples of such mechanisms. Primarily, we analysed all variables from these evaluations; however, as some of the variables had a low communality, they could be excluded without any loss of information. Lastly, we knew from a clinical point of view that a factor indicating cardiac function was important, and thus the NT-proBNP concentration was also included.

In a second step, Structural Equation Modelling (SEM) ad modum LISREL was used to explore associations or structural relations/effects between the underlying factors. The SEM approach allows researchers to determine if there are direct as well as indirect relationships between independent and dependent variables [71].

In the third step, the effect of selenium concentration *at baseline* on the model structure was analysed. Finally, linear structural relationships and factor structures were combined into one comprehensive model. Goodness-of-fit of the SEM model was examined with Chi-Square, the Root Mean Square Error of Approximation (RMSEA), and the Comparative Fit Index (CFI). A non-significant Chi-Square, a RMSEA <0.06 and a CFI ≥0.95 indicate a good model fit [71].

In the *first step* of the present analysis, 13 different biomarkers (i.e. copeptin, MR-proADM, TNFr1, TNFr2, osteoprotegerin, osteopontin, endostatin, galectin, TIMP1, cathepsin S, GDF-15, MMP-1, and NT-proBNP) were factored into latent variables or factors. The factor and modelling analyses were based on data from the *initial* measurements and thus reflect the natural variation of the biomarkers in the total group at baseline.

*Then*, the factors identified were used in reliability analyses and comparisons of groups between the first and second occasion 48 months later.

Finally, the relative predictive effects of selenium status and NT-proBNP values (as independent variables) on the subjects age (as dependent variable) were analysed in a regression model. The value was called “CV age” and represents the optimal linear predictive power of selenium and NT-proBNP.

## Results

### Data reduction and modelling analyses

Confirmative Factor Analyses (CFA) were, in the first step, performed on data from the initial measurement occasion. In the final analysis, three factors, viz. “Inflammation”, “Oxidative Stress”, and “Fibrosis” were extracted. The common variance between TNF receptor1, TNF receptor2, osteoprotegerin, and osteopontin was explained by the factor “Inflammation”, the common variance between copeptin, and MR-proADM was explained by the factor “Oxidative Stress”, and the common variance between endostatin, galectin 3, TIMP 1, cathepsin S, GDF-15 and MMP-1 was explained by the factor “Fibrosis”. The fit of the factor structure was good with a RMSEA of 0.042, and a Critical Fit Index (CFI) of 0.96, indicating that 96 percent of the co-variances between the 13 biomarkers were explained by the three factors. The construct reliability for “Inflammation” was 0.63, 0.70 for “Oxidative Stress”, and 0.63 for “Fibrosis”. The index presents the proportion of true variance explained by the factor, and values higher than 0.60 indicate a high reliability.

In the *second* step, SEM ad modum LISREL was used to model or explore associations or structural relations/effects between the underlying factors “Inflammation”, “Oxidative Stress”, and “Fibrosis”. To the hypothesized basic model, a factor “Myocardium” representing NT-proBNP (indicating myocardial wall tension) was added. In the model also, a second-order factor “Inflammation/Oxidative Stress” combining the factors “Inflammation” and “Oxidative Stress” was hypothesized.

When scrutinizing the correlation matrix of the variables, we found that half of the biomarkers at the first occasion were slightly, but significantly, related to the age of the participant (the mean correlation was 0.25 with a range from 0.18 to 0.37). This relation may inflate the correlations of the input matrix and affect the factor relations of the SEM model. This age effect was controlled by means of partial correlation analysis, and the corrected correlation matrix was compared with the uncorrected one. From the comparison, we found that the age effect was only marginal, and 98 percent of the variable relations remained the same. Accordingly, our use of the original matrix in our analyses was valid.

Finding relations between age and several of the biomarkers was expected and has also been reported elsewhere. There was a small but significant relation ( – 0.13, *p* = 0.001) between selenium concentration (at the first occasion) and age. This has been previously reported in the literature, but in a much younger population [8]. To compensate for the age relation, we created a *quotient* between selenium concentration and age. This independent factor here called “Sel/age” is related 0.84 to selenium concentration, and  – 0.64 to age.

In the *third* step, this quotient Sel/age was included as an independent factor. By means of this factor we could analyse if and to what extent selenium influences the factors of the basic model.

**Fig. 3:**
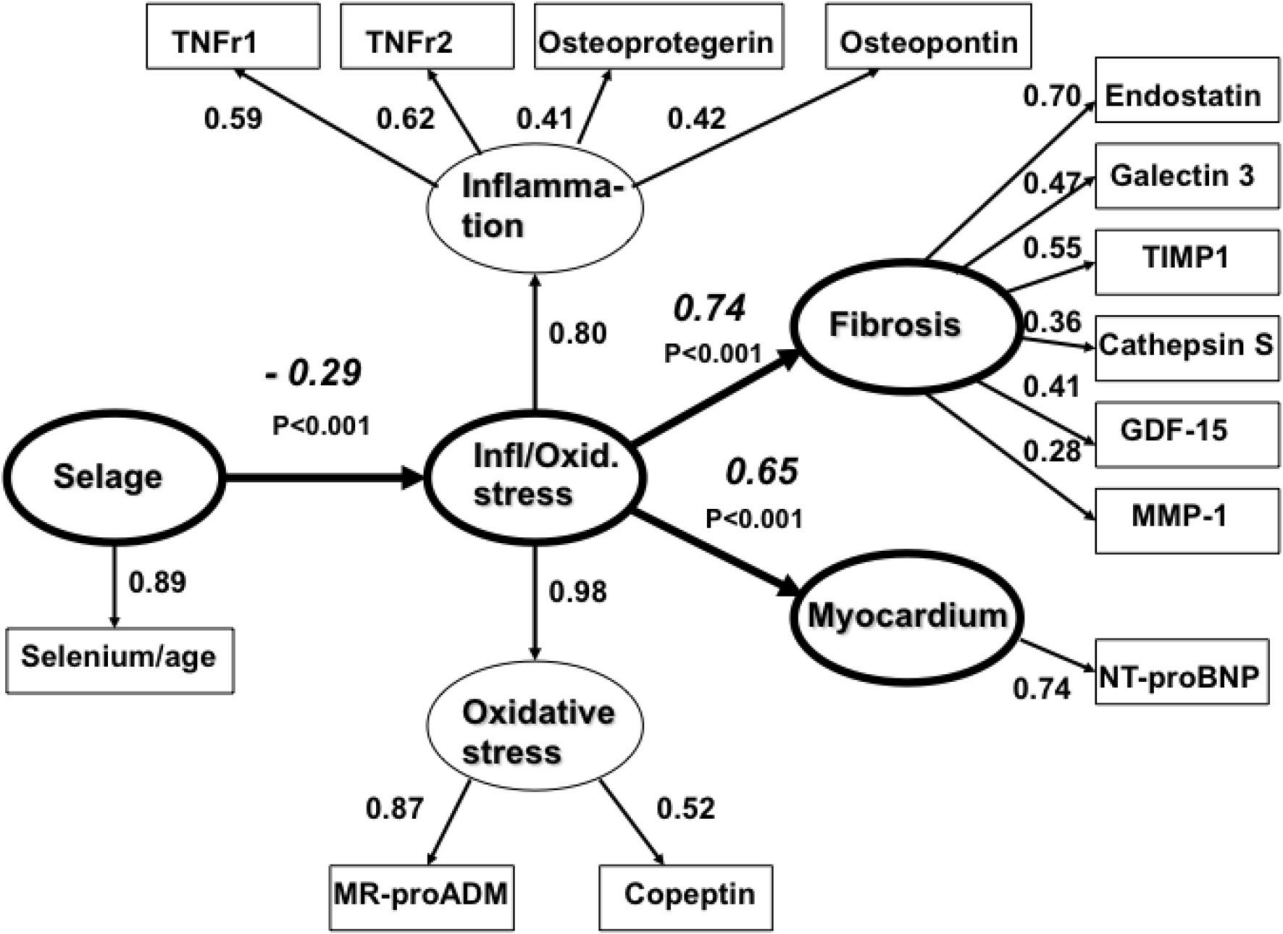
Structural equation model of the relations between Selenium (Selenium/age), the second-order factor Infl/Oxid. Stress, Fibrosis and Myocardium. Ellipses depict latent variables or factors and squares manifest or measured variables. All effects (Beta-values italicized) and factor loadings are significant (*p* < .001). Model fit: Chi-square = 56.43, df = 57, *p* = 0.497, RMSEA = 0.000, CFI = 0.99

The final model is presented in Fig. 3. As can be seen from the model (Fig. 3), the first-order factors “Inflammation” and “Oxidative Stress” form a second-order factor “Infl/Oxidative Stress”. The weights of the first-order factors on the second-order factor are high and about the same (0.98, and 0.80). As hypothesized, the factor “Infl/Oxidative Stress” has strong influences on the factors “Fibrosis” (β = 0.74; *p* < 0.001) and “Myocardium” (β = 0.65; *p* < 0.001). Finally, the model shows that the independent factor “Sel/age” significantly influences the second-order factor “Infl/Oxidative Stress” (β = – 0.29; *p* < 0.001). All indirect effects (β weights) of “Sel/age” are significant: “Sel/age” → “Inflammation”,  – 0.23, (*p* = 0.001), “Sel/age” → “Oxidative Stress”,  – 0.29, (*p* < 0.001), “Sel/age” → “Fibros”,  – 0.21, (*p* = 0.003), and “Sel/age” → “Myocardium”,  – 0.19, (*p* = 0.007). This means that the second-order factor “Infl/Oxidative Stress”, representing the factors “Inflammation” and “Oxidative Stress”, acts as a mediating factor between the independent selenium factor “Sel/age” and the dependent factors “Fibrosis” and “Myocardium”. The fit of the presented model is almost perfect (Chi-Square = 56.43, df = 57, *p* = 0.497, RMSEA = 0.000, CFI = 0.99), meaning that it explains almost all of the true variance between the manifest or measured variables.

As noted above, the factors “Inflammation”, “Oxidative Stress”, and “Fibrosis” have high construct reliability. Their test–retest reliabilities are also high. The correlation between test and retest for “Inflammation” was 0.57 (*p* < 0.001), for “Oxidative Stress”0.67 (*p* < 0.001), and for “Fibrosis”0.76 (*p* < 0.001). This means that 32, 45, and 45 percent, respectively, of the factor variance after 48 months can be explained by the factor variance at the initial test occasion.

### Changes after intervention

**Fig. 4:**
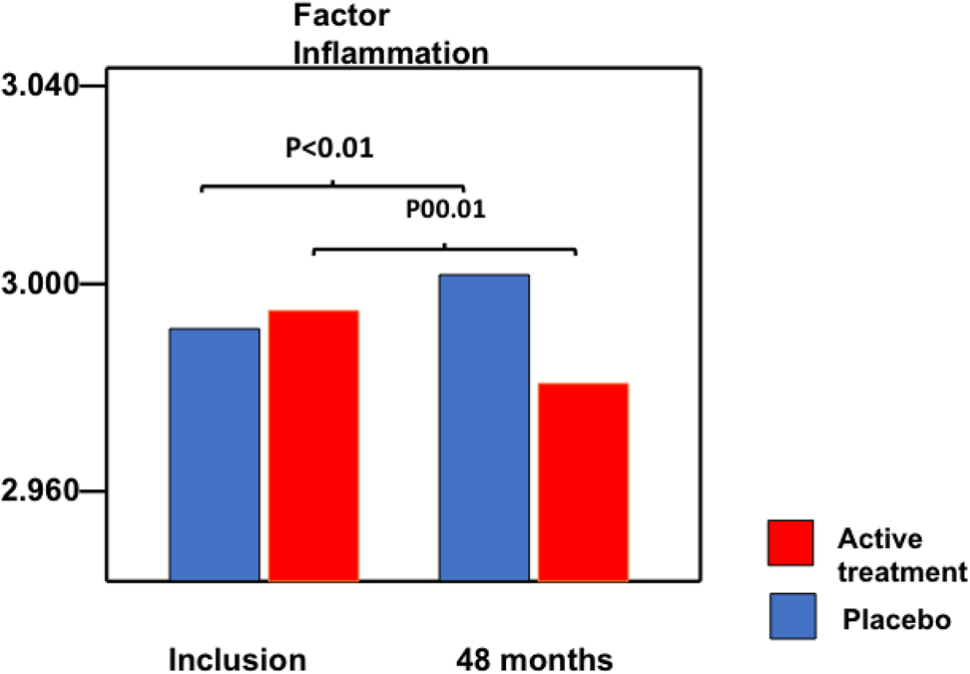
The obtained changes in factor weights between the factors “Inflammation” before and after intervention with selenium and coenzyme Q_10_

Changes in the active treatment and the placebo groups were evaluated over the 48 months. For the factor “Inflammation”, a significant increase between inclusion and 48 months was noted in the placebo group (T =  – 4.1; *p* < 0.001), whereas in the active treatment group, a significant decrease was noted (T = 2.6; *p* = 0.010) (Fig. 4). Upon evaluation of the effect on the factor “Fibrosis”, it was revealed that in the placebo group, no significant difference was seen between inclusion and after 48 months (T = 1.2; *p* = 0.23); however, in the active treatment group, a significant decrease was noted (T = 8.1; *p* < 0.001). In the placebo group, the factor “Oxidative Stress”, increased significantly (T =  – 2.21; *p* = 0.03), whereas in the active treatment group, there was no significant difference between inclusion and 48 months (T = 0.39; *p* = 0.70).

NT-proBNP is a critical marker of the heart function, and its reference range in healthy individuals increases with increasing age after about 50 years of age [72, 73]. In the present population (age 79–88), selenium supplementation was associated with reduced NT-proBNP-levels. In a multiple regression analysis, using data obtained at baseline, we examined the relative predictive effects of selenium and NT-proBNP (as independent variables) on the subjects age (as dependent variable). From the analysis, an algorithm predicting the functional age of the heart or “CV age” (“CV age” = 82.185–**0.185** * selenium + **0.354** * NT-proBNP) was evaluated in the cohort. Both the weight for selenium ( – **0.185**, *t* = – 2.875, *p* = 0.004) and the weight for NT-proBNP (**0.354**, *t* = 5.502, *p* < -0.001) as well as the fit of the total regression model (ANOVA: Mean Square = 161.570, F-ratio = 21.841. *p* < 0.001) were significant.

**Fig. 5:**
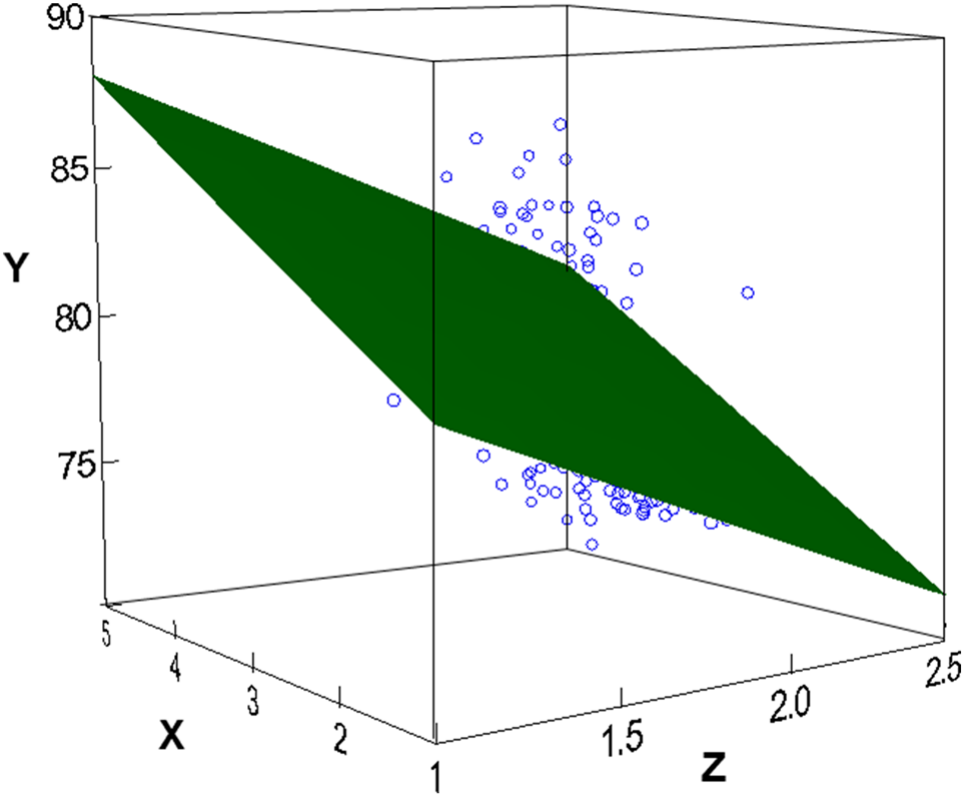
A linear regression smoothing plane optimizing the fit of data points in a three-dimensional space. The figure is based on the regression equation predicting ”CV age” from logarithmized values of NT-proBNP and Selenium. X = LogNT-proBNP, Y = ”CV age”, and Z = logselenium

Figure 5 shows a linear regression smoothing plane optimizing the fit of data points in a three-dimensional space.

As can be seen from the figure, the highest value of NT-proBNP, in combination with the lowest value of selenium predicts an”CV age” of about 88 years (A), and the lowest value of NT-proBNP in combination with the highest value of selenium predicts a an”CV age” of about 72 years (B). In the present population of elderly individuals with a suboptimal selenium status, the correlation between real age and”CV age” is as low as 0.40 (*p* < 0.001). However, it should be taken into consideration that the range of true age of the subjects is 79–88 years. Furthermore, it is apparent that extrapolations of this relation to populations with optimal selenium intake (> about 100 ug/day), for instance, the cohort in the Framingham study, should not be done.

## Discussion

The main findings of the randomized double-blind placebo-controlled intervention with selenium and CoQ_10_ in elderly Swedes low in selenium and coenzyme Q_10_ reviewed here, were a significantly increased systolic heart function, a reduced myocardial wall tension, and a reduced CV mortality. Based on these findings, a series of secondary analyses to explore some of the underlying mechanisms for the positive clinical results were conducted. Through these analyses, we found evidence for a causal relation between the intervention and changes in a series of biomarkers, including expression of circulating microRNAs.

To further examine the relationships between the impacts on different biomarkers, we undertook an overall analysis of our observations using the SEM approach, which is considered a robust technique regarding causal inferences. The presented SEM analysis provides mechanistic a cross-validation of the findings from the classical RCT design by first developing a model based on data obtained at baseline (Fig. 3), and second, using the follow-up data, we also examined the impact of the intervention in the model (Figs. 4 and 5) in studies using only the RCT approach statistically significant, but clinically insignificant differences, can be generated.

From the SEM analysis, we conclude that the second-order factor “Infl/Oxidative Stress”, representing the factors Inflammation and Oxidative Stress, acts as a mediating factor between the apparently independent selenium factor Sel/age, and the dependent factors, fibrosis and myocardium. Identification of these independent and dependent factors is based on determinations before the intervention, that there is a well-known inter-relationship between age and inflammation is illustrated by the concept of “Inflammaging” presented by Franceschi in 2007 [74].

The multiple regression analyses indicate that NT-proBNP and selenium together, but independently of each other, may predict a functional age, “CV age”, for the present subjects. While the NT-proBNP-value may act to increase this theoretical age, increasing selenium levels reduces the “CV age”.

The results obtained support the notion that selenium status is a central determinant for several processes in the body, as seen in the present population with a relative deficiency in selenium/coenzyme Q_10_. Further, the supplementation has verified effects on inflammation, on oxidative stress, on fibrosis and also directly on the myocardium.

The reported decreased levels of both the von Willebrand factor, and PAI-1, resulting from the intervention, indicate that endothelial dysfunction, as an early sign of vascular damage, is alleviated by the intervention.

As clinical results evaluated also include “end-stage” results in a long process (cardiovascular mortality and experienced health-related quality of life [75]), the early signs of impairment such as endothelial dysfunction are important for the prevention.

The endpoint CV mortality is the most definitive endpoint to be used when evaluating effects of intervention on cardiac health. The four years of intervention with selenium and coenzyme Q_10_ reduced CV mortality not only after five years of follow-up but also eight years after termination of the supplementation. This long-lasting effect may indicate beneficial structural changes due to the intervention.

As the actual genesis to the CV mortality in the present study differs between heart failure, myocardial infarction, fatal arrhythmias, including cerebrovascular events, we suggest that the effect of the intervention could be found in basic vascular mechanisms, e.g. endothelial dysfunction and inflammation.

There are several studies in the literature where either selenium or coenzyme Q_10_ was given as monotherapy. Rees et al. presented a Cochrane systematic review to elucidate possible effects of intervention with selenium on prevention of cardiovascular disease [14]. Their conclusion was that no data included in their review could support the supplementation with selenium. However, an important fact in their study was that out of the 19,715 participants, 18,415 (93.4%) came from a US-based population, a population that had high baseline selenium [10, 11] and thus, it is not surprising that supplementation with selenium did not give significant effects. Underscoring this is our finding that the tertile lowest in plasma selenium concentration in terms of CV mortality benefited most from the active intervention [40].

There are some studies where interventions have been performed using a multi-supplement approach, where selenium and coenzyme Q_10_ have been part of the mixture given. Improved cardiac function and perceived quality of life were reported by Witte et al. in a study of patients with heart failure using capsules containing 14 different micronutrients, including coenzyme Q_10_ and selenium, or placebo [76].

Even if our main study had a relatively small sample size, the follow-up period was long, up to 12 years, and the secondary analyses consisted of several evaluated variables. The significant results obtained from the many different evaluations confirms that obvious effects do occur from the intervention, and it seems that the lower the selenium intake, the more powerful results of the intervention.

It should be emphasized that the baseline selenium status in the elderly population of the present Kisel study was suboptimal or inadequate, implying that extrapolation of the results to other populations with a higher selenium intake should be done with care.

To re-examine our observations, we would propose a large-scale long-term intervention study in another population with comparable baseline selenium values and use of the same type of intervention.

## Conclusion

An intervention study for four years with selenium and coenzyme Q_10_ combined performed in elderly community-living Swedish subjects showed substantial and long-lasting clinical effects that included improved cardiac function and reduced cardiovascular mortality. We have here given a short summary of some of the most important findings, including the findings on biomarkers of inflammation, oxidative stress and fibrosis. From these findings, it could be demonstrated that supplementation with selenium and coenzyme Q_10_ could significantly reduce the level of these processes. To validate these results and to further explore possible mechanisms behind these clinical results, we conducted a secondary analysis based on previously published results on a series of biomarkers measured at baseline and during the follow-up period. By applying Structural Equation Modelling (SEM), we identified a model of possible mechanisms. From the model, it could be deduced that the lower the selenium concentration of the individual, the higher was inflammatory activity as well as biomarkers on oxidative stress. Furthermore, suboptimal selenium status was accompanied by a higher level of fibrosis and increased myocardial wall tension which in turn influences the risk of cardiovascular mortality. The model was validated using data obtained after 48 months of intervention.

Thus, the model shows that in individuals with low selenium and coenzyme Q_10_ concentration, supplementation with the two substances makes a difference in terms of inflammation, oxidative stress, fibrosis and cardiovascular risk.

## Data Availability

Under Swedish Law, the authors cannot share the data used in this study and cannot conduct any further research other than what is specified in the ethical permissions application. For inquiries about the data, researchers should first contact the owner of the database, the University of Linköping. Please contact the corresponding author with requests for and assistance with data. If the university approves the request, researchers can submit an application to the Regional Ethical Review Board for the specific research question that the researcher wants to examine.

## Abbreviations

ACEI: ACE inhibitors
ARB: Angiotensin receptor blockers
CV: Cardiovascular
EF: Ejection fraction
ECG: Electrocardiogram
Hs-CRP: High-sensitivity analysis of C-reactive protein
IHD: Ischaemic heart disease
NYHA class: New York heart association functional class
PAI-1: Plasminogen activator inhibitor 1
SD: Standard deviation
SEM: Structural equation modelling
vWf: Von Willebrand factor
